# An In-Line Fiber Optic Fabry–Perot Sensor for High-Temperature Vibration Measurement

**DOI:** 10.3390/mi11030252

**Published:** 2020-03-01

**Authors:** Dong Chen, Jiang Qian, Jia Liu, Baojie Chen, Guowen An, Yingping Hong, Pinggang Jia, Jijun Xiong

**Affiliations:** Science and Technology on Electronic Test and Measurement Laboratory, North University of China, Taiyuan 030051, China; dongchennuc@163.com (D.C.); QJiangnuc@163.com (J.Q.); 18734920710@163.com (J.L.); chenbaojie_nuc@163.com (B.C.); anguowen@nuc.edu.cn (G.A.); hongyingping@nuc.edu.cn (Y.H.); xiongjijun@nuc.edu.cn (J.X.)

**Keywords:** fiber optic sensor, Fabry–Perot, high temperature, vibration measurement

## Abstract

An in-line fiber optic Fabry–Perot (FP) sensor for high-temperature vibration measurement is proposed and experimentally demonstrated in this paper. We constructed an FP cavity and a mass on single-mode fibers (SMFs) by fusion, and together they were inserted into a hollow silica glass tube (HST) to form a vibration sensor. The radial dimension of the sensor was less than 500 μm. With its all-silica structure, the sensor has the prospect of measuring vibration in high-temperature environments. In our test, the sensor had a resonance frequency of 165 Hz. The voltage sensitivity of the sensor system was about 11.57 mV/g and the nonlinearity was about 2.06%. The sensor could work normally when the temperature was below 500 °C, and the drift of the phase offset point with temperature was 0.84 pm/°C.

## 1. Introduction

High-temperature vibration sensors are essential for the maintenance and fault detection of facilities and equipment in many fields, such as aerospace, oil and gas pipeline transportation, volcanic seismic activity detection, coal ore mining, and other fields, as well as the health monitoring of structures [1,2,3,4,5]. Fiber optic vibration sensors have been proven to have good application prospects in high-temperature measurement in past research, and they have the advantages of small size, high accuracy, and resistance to electromagnetic interference [6,7,8,9,10,11]. The fiber optic vibration sensors are mainly based on fiber Bragg gratings (FBGs), Mach–Zehnder interferometers, or Fabry–Perot (FP) interferometers. Of these, the FP interferometer can be easily formed in small structures, and it has the advantages of stability and is easy to be demodulated [12,13,14,15,16]. Meanwhile, among the fiber optic vibration sensors, the in-line fiber optic vibration sensor is particularly small, can be embedded in the structure for detection, and has the prospect of multi-point distributed measurement and combination with other types of sensors.

In recent years, there has been some research and exploration on in-line fiber optic sensors [17,18,19,20]. Wang et al. constructed a Fabry–Perot interferometer by splicing a section of hollow silica glass tube (HST) between two optical fibers to detect high-intensity focused ultrasound [18]. Liao et al. constructed two micro-tapers along the longitudinal direction in a single-mode fiber (SMF), and applied an annealing at 1000 °C to stabilize the temperature measurement [19]. Fang et al. constructed an FP cavity in cascaded microbubbles to enhance the sensitivity of strain measurement [20]. In the research on vibration sensors, in-line fiber optic sensors usually measure vibration by constructing a micro-cantilever beam and micro-mass [21,22,23,24,25,26,27,28]. Lu et al. etched a micro-beam in the cladding of a single-mode fiber by chemical etching, then spliced another section of SMF. They demodulated the vibration signal by collecting the light intensity changes caused by vibration, and realized the demodulation of vibration signals above 5 kHz [26]. Zhang et al. wet etched a mass in the cladding of an SMF, and used an FP cavity and fiber Bragg grating, respectively, to detect the vibration signal [27,28]. However, a sensor fabricated by wet etching has a fragile structure and requires a long manufacturing cycle, and the thinning of the optical fiber may cause loss of the light intensity.

In this paper, we present an in-line fiber optic FP vibration sensor for high-temperature vibration measurement. We constructed an FP cavity and a mass on SMFs, where the FP cavity was constructed by fusing an HST at the end faces of two optical fibers, and the mass was constructed by fusing another type of HST on one of the optical fibers. When vibration occurs, the vibration of the mass causes changes of the FP cavity length, and the changes in cavity length can directly respond to changes in vibration. On these bases, the light intensity demodulation system and the experimental setup were established, and the sensing characteristics were tested and analyzed. The sensor has the advantages of high light intensity, small size, simple manufacturing process, and low cost. With its all-silica structure, the sensor has the prospect of measuring vibration in high-temperature environments.

## 2. Fabrication and Principle

**Figure 1:**
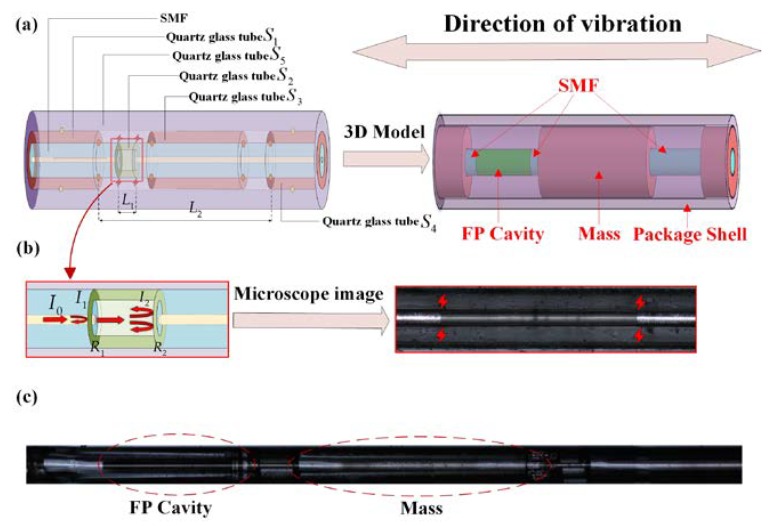
In-line fiber optic sensor for vibration measurement: (**a**) structural configuration and three-dimensional (3D). (**b**) Fabry–Perot (FP) cavity. (**c**) the microscope image of the sensor. SMF: single-mode fiber.

The proposed configuration of the in-line fiber optic FP sensor for vibration measurement is shown in Figure 1a. The sensor consists of an FP cavity, two single-mode fibers, a mass, and a package shell. In this shell, the end faces $R_{1}$ and $R_{2}$ of the two optical fibers and the hollow silica glass tube $S_{2}$ form an FP cavity, as shown in Figure 1b. The hollow silica glass tubes $S_{1}$, $S_{3}$, and $S_{4}$ are sequentially welded on the optical fibers, among which the HST $S_{3}$ is used as a mass and $S_{1}$ and $S_{4}$ are used to match the outer diameter. The hollow silica glass tube $S_{5}$ is tightly welded to $S_{1}$ and $S_{4}$, and together they form the package shell of the sensor. Multi-beam interference that occurs in the FP cavity can be used to measure the cavity length. The displacement of the mass block caused by the inertial force can make the fiber stretch and then change the length of the FP cavity. The package shell can protect the internal structure of the sensor and restrict the vibration of the mass in the radial direction.

### 2.1. Sensor Fabrication

The sensor consists of three different sizes of hollow silica glass tubes welded to two single-mode fibers (AS19/125/155G, Fiberguide Industries, Ltd., Stirling, NJ, USA). The parameters of all HSTs and fibers used in the fabrication are shown in Table 1. The HSTs $S_{1}$, $S_{3}$, and $S_{4}$ all have an inner diameter of 135 μm and an outer diameter of 340 μm, in which $S_{3}$ as a mass can be cut according to requirements to obtain different quality masses. The HST $S_{2}$ has an inner diameter of 65 μm and an outer diameter of 125 μm, and its outer diameter matches the optical fiber. Different lengths of the FP cavity can be obtained by cutting $S_{2}$. The HST $S_{5}$ has an inner diameter of 350 μm and an outer diameter of 450 μm. Adjusting the inner and outer diameter of $S_{5}$ can improve the mechanical strength of the sensor.

**Figure 2:**
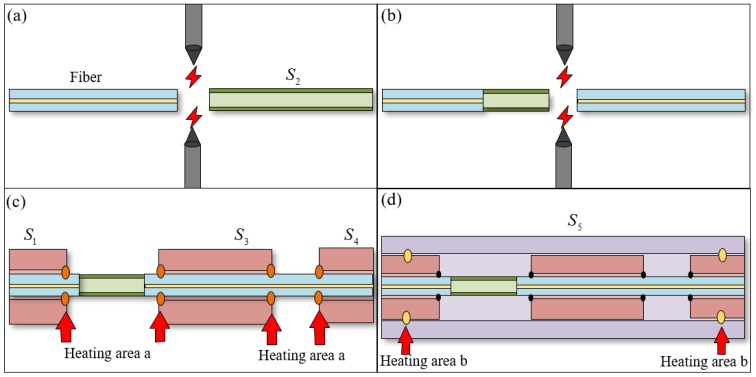
Fabrication process of the proposed in-line fiber optic sensor. (**a**) The welding of the SMF and the HST $S_{2}$ , (**b**) The welding of the FP cavity, (**c**) The welding of the HSTs $S_{1}$, $S_{3}$, and $S_{4}$, (**d**) The welding of the package shell.

The manufacturing process of the sensor includes the fabrication of the FP cavity, the welding of the mass, and the welding of the package shell. As shown in Figure 2a, the SMF and the HST $S_{2}$ are cut flat with a cutter (CT30, Fujikura, Ltd., Tokyo, Japan) and placed into a commercial fusion splicer (62S, Fujikura, Ltd, Tokyo, Japan) for discharge welding, and the discharge parameters are 5 bit (about 4.3 W) and 200 ms. By controlling the fusion splicing parameters, the mechanical strength of the FP cavity can be ensured without loss of light intensity. As shown in Figure 2b, the HST $S_{2}$ welded on the optical fiber is cut again, and the cut $S_{2}$ is spliced with another fiber; the discharge parameters are exactly the same as in Figure 2a. At this point, the fabrication of the FP interference cavity is finished. As shown in Figure 2c, the HSTs $S_{1}$, $S_{3}$, and $S_{4}$ are inserted on the optical fiber, respectively, then a carbon dioxide laser fusion splicer (LZM–110, Fujikura, Ltd, Tokyo, Japan) is used to perform laser welding at the positions shown in the figure. The welding parameters are 342 bit (about 10.2 W) and 800 ms. Adjusting the position of the heating area can reduce the radial shaking of the optical fiber. Finally, the inner part of the sensor is sleeved into the large hollow silica glass tube $S_{5}$, and $S_{1}$, $S_{4}$, and $S_{5}$ are welded by the carbon dioxide laser fusion splicer. The welding parameters are 425 bit (about 12.8 W) and 800 ms. $S_{1}$, $S_{4}$, and $S_{5}$ constitute the package shell of the sensor, as shown in Figure 2d. Table 2 lists the welding parameters for all welds, where heating area A is a random point on the contact surface between the HSTs and the SMF, and heating area B is a random point on the contact surface between the HSTs.

The parameters of each HST and the welding parameters of the fusion splicer can be changed according to the situation. Adjusting the welding parameters can achieve stable welding without loss of light intensity. Adjusting the inside and outside diameter of the HST and the position of each welding point can reduce the effects of vibration outside the axis. The fabrication process of the proposed in-line fiber optic sensor is simple and has the advantage of a short manufacturing cycle. The formed sensor is small in size and can be embedded as a probe into an object to detect the vibration in a specific direction. The microscope image of the fabricated in-line fiber optic vibration sensor is shown in Figure 1c. It can be seen that the HSTs $S_{1}$, $S_{4}$, and $S_{5}$ are closely attached to ensure the integrity of the entire sensor head, while $S_{3}$, which is tightly welded to the optical fiber, can move freely as a mass. In addition, the interference spectrum of the FP cavity is measured by a spectrum analyzer, and the contrast can reach up to 20 dB.

In summary, the fabrication process of the proposed in-line fiber optic sensor is simple and has the advantage of a short manufacturing cycle. The formed sensor is small in size and can be embedded as a probe into an object to detect the vibration in a specific direction. As long as the parameters of each HST and the welding parameters of the fusion splicer can be changed, the sensor can be applied to different vibration environments.

### 2.2. Operation Principle

According to Figure 1a, the structural configuration can be simplified as a spring mass system. The change of cross-sectional area caused by stretching can be ignored. According to Hooke’s law, the relationship between the change in cavity length (Δl) and the acceleration (a) is as follows:

(1) $$\Delta l=\frac{maL_{1}}{EA}$$

where E is the Young’s modulus of silica glass, A is the cross-sectional area of $S_{2}$, m is the mass of $S_{3}$, $L_{1}$ is the length of $S_{2}$, and the initial cavity length ($l_{0}$) is $l_{0}=L_{1}$. 

According to the small deflection theory, the resonance frequency (ω) of the sensor at this point is:

(2) $$\omega =\frac{1}{2\pi }\sqrt{\frac{E\pi d^{4}}{64L_{{}_{2}}^{3}m}}$$

where d is the diameter of the optical fiber and $L_{2}$ is the distance between the two fixed ends of the optical fiber, as shown in Figure 1a. 

According to Equations (1) and (2), when designing the sensor, we can change the length of the original cavity and the size of the mass to obtain the required sensitivity and resonance frequency.

Based on the principle of multi-beam interference, when the phase of the reflected light is at the phase offset point ($\phi_{m}$) and the output optical power at the phase offset point is $I_{m}$, the output optical power ($I_{out}$) and the phase change ($\phi_{a}\left(t\right)$) caused by vibration have the following relationship [29]:

(3) $$I_{out}=I_{m}+K_{m}\cdot \phi_{a}\left(t\right)$$

where $\phi_{a}\left(t\right)=4\pi n\Delta l/\lambda$, n is the refractive index of the air, and $K_{m}$ is the slope of the output optical power as a function of phase at the phase offset point. 

According to Equation (3), it can be seen that when the phase offset point is determined and stable, the output optical power ($I_{out}$) is linear with the change of cavity length (Δl), and when there is no vibration, the output optical power is the output optical power at the phase offset point ($I_{m}$). According to Equations (2) and (3), the relationship between acceleration (a) and output voltage ($V_{out}$) when loading vibration can be written as:

(4) $$V_{out}=V_{m}+K_{m}GR\frac{4\pi nmL_{1}}{\lambda EA}\cdot a$$

where $V_{m}$ is the direct current (DC) voltage output at the phase offset point, and G,R are gain and response factors of photoelectric detection, respectively. 

According to Equation (4), it can be seen that the output voltage and acceleration have a linear relationship. However, when the external temperature rises, the change in cavity length will be affected by other factors due to the thermal expansion of silica. The optical power at the phase offset point ($I_{m}$) and the slope of the output optical power as a function of phase at the phase offset point ($K_{m}$) will change, but we can still adjust the wavelength of the laser to re-determine the position of the phase offset point.

## 3. Results and Discussion

**Figure 3:**
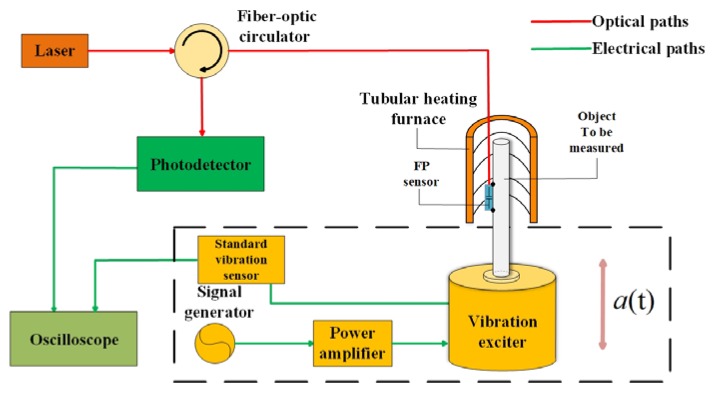
The schematic diagram of the sensor test system.

The schematic diagram of the sensor test system is shown in Figure 3. The experimental setup consisted of a vibration exciter, a standard vibration sensor, a tubular heating furnace, and a light intensity demodulation system. The in-line fiber optic sensor was attached to the surface of the object to be tested (quartz rod) with high-temperature glue (YK–8927, Yikun glue, Macheng, China), and together put into the tubular heating furnace (GSL–1100X–S, HF kejing, Hefei, China), and the quartz rod was connected to the vibration exciter (TV 50101, Tira, Thuringen, Germany). The standard vibration sensor was integrated in the vibration exciter as part of its calibration system, which could detect the vibration generated by the sine generator of the exciter. At the same time, in the intensity demodulation system, the light generated by the tunable laser (GM82009, Guilin GM Technology Industry Ltd, Guilin, China) was transmitted to the sensor through the coupler (1310/1550–SSC–1*2), and the reflected light was converted into an electrical signal through the photodetector (Model 2053, New Focus, San Jose, CA, America), which was connected to the oscilloscope for display and storage.

It should be clearly noted that in our test the length of the mass in the sensor was about 5 mm, and $L_{2}$ was set to 10 mm. The quality of the mass can be estimated as 0.8 mg, approximately. According to Equation (2), the resonance frequency of the sensor can be calculated as ω=164.5094  Hz.

**Figure 4:**
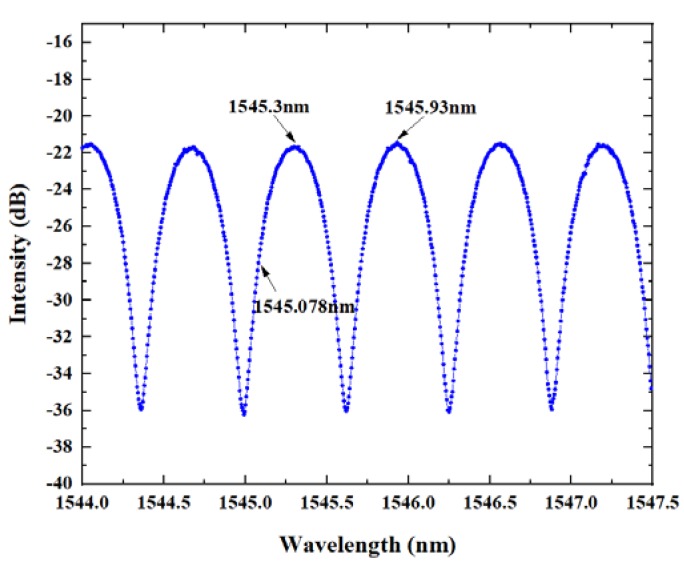
The reflected spectrum of the FP cavity.

In order to determine the phase offset point at room temperature, a tunable laser was used for wavelength scanning, and the adjustable range of the laser wavelength was from 1525 to 1565 nm. When the wavelength scanning ranged from 1544 to 1547.5 nm, the reflected signal spectrum of the FP cavity in the sensor is shown in Figure 4. As shown in the figure, the wavelength of the phase offset point was 1545.078 nm, and the cavity length of the Fabry cavity could be calculated as $l=\lambda_{1}\lambda_{2}/\left[2\left(\lambda_{1}-\lambda_{2}\right)\right]$, where $\lambda_{1}=1545.93\;\mathrm{nm}$ and $\lambda_{2}=1545.3\;\mathrm{nm}$, with the initial cavity length of l=1.89 mm. And the initial free spectral range (Δv) is determined by $\Delta v=\lambda^{2}/2nl$, λ is the wavelength of the phase offset point, n is the refractive index of the air, l is the initial cavity length, Δv=6.31 nm.

**Figure 5:**
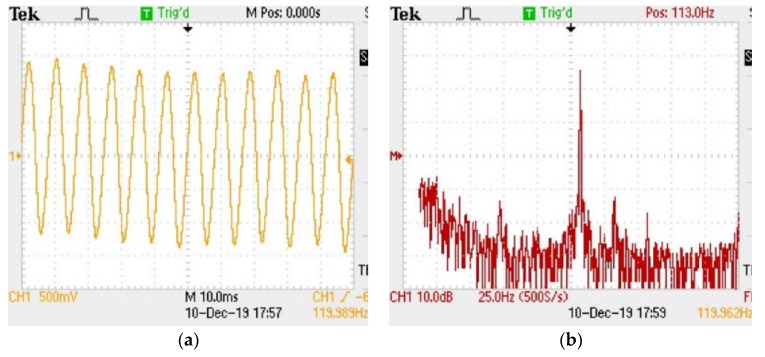
The oscilloscope output of the sensor under 120 Hz and 10 g at room temperature: (**a**) waveform of vibration. (**b**) fast Fourier transform spectrum of the waveform.

The performance of the sensor was tested at room temperature. The time history and the corresponding frequency spectrum of the sensor under the vibration excitation with the frequency of 120 Hz and acceleration of 10 g are shown in Figure 5a and b, respectively. It can be seen in Figure 5a that the sensor could output a stable sinusoidal vibration signal at 120 Hz. According to the peak–peak voltage value of the waveform, the voltage sensitivity of the system could be calculated to be about 11.57 mV/g when the gain of the photodetector in the light intensity demodulation system was 1000. It can be seen from Figure 5b that the sensor had a frequency response at the frequency of 119.96 Hz, which is in good agreement with the frequency of the vibration exciter.

**Figure 6:**
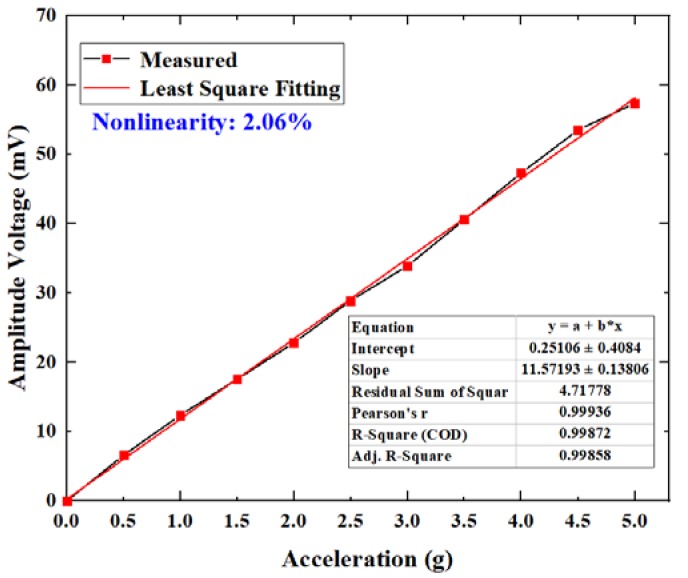
The nonlinearity curve of the sensor.

At the same time, at the frequency of 120 Hz, we slowly increased the magnitude of acceleration excitation from 0 to 5 g, and recorded a group of data every 0.5 g. The amplitude voltage values and the least-square fitting of the data are shown in Figure 6. It can be seen that the output voltage was proportional to the measured acceleration and the calculated nonlinearity was approximately 2.06%. The nonlinearity may have been affected by environmental noise and other factors, resulting in a large change in DC output.

**Figure 7:**
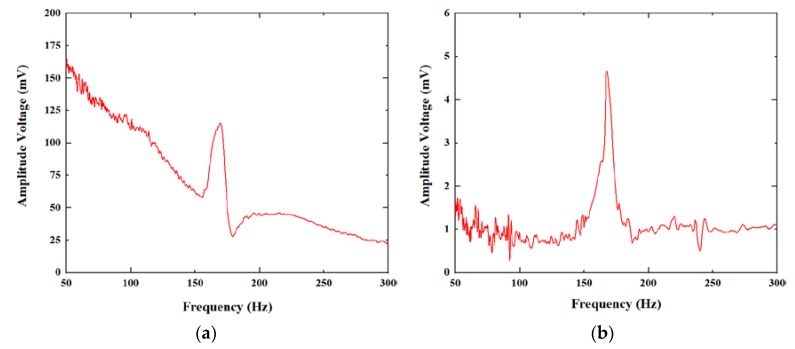
The frequency response of the sensor: (**a**) the axial direction. (**b**) the radial direction.

Then, an acceleration excitation with amplitude of 10 g was applied to the sensor in the axial and radial directions, and the frequency was swept from 50 to 300 Hz. The response is shown in Figure 7. The axial and radial directions showed similar resonance frequencies, both around 165 Hz, and the voltage value of radial vibration was extremely small compared to the voltage value of axial vibration. 

**Figure 8:**
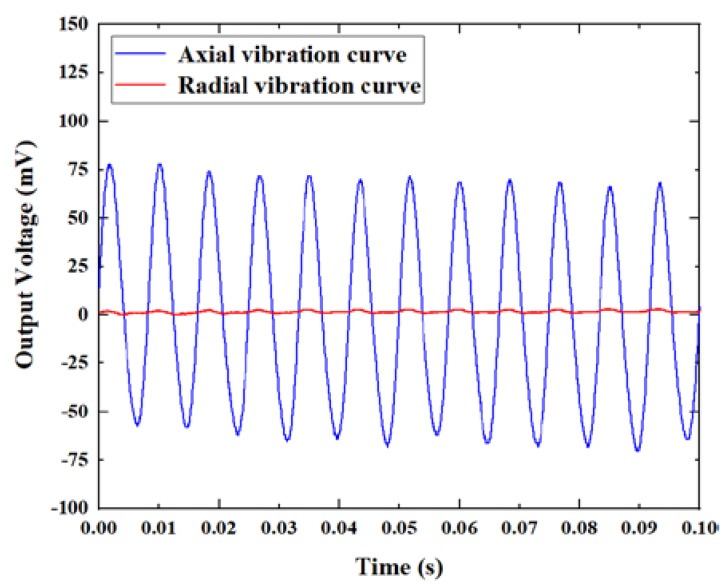
The axial and radial vibration response of the sensor.

In order to further determine the relationship between the axial vibration and the radial vibration, we applied 6 g acceleration at a frequency of 120 Hz, and simultaneously recorded the axial and radial vibration signals. The output signal of the sensor under the axial vibration excitation and the radial vibration excitation is shown in Figure 8. It can be seen that the sensitivity of the radial vibration was much lower than that of the axial vibration. The sensitivity of the axial vibration was approximately 60 times that of the radial vibration. This is because we reduced the radial jitter range of the fiber by adjusting the parameters of the heating point. Adjusting the position of the heating point could further reduce the sensitivity of radial vibration and improve the accuracy of the axial vibration measurement of the sensor.

**Figure 9:**
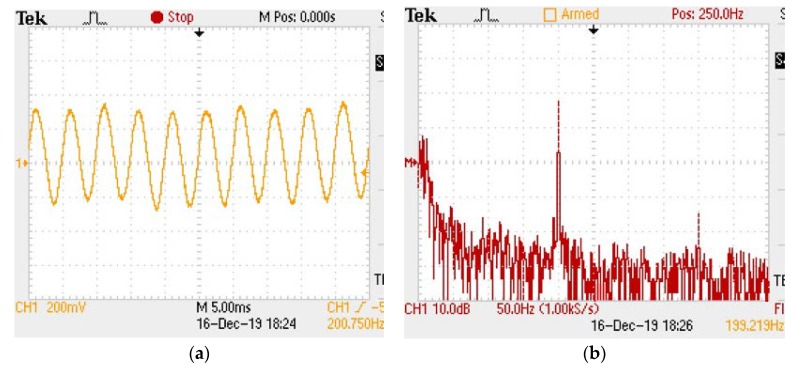
The oscilloscope output of the sensor under 200 Hz and 10 g at 500 °C: (**a**) waveform of vibration; (**b**) fast Fourier transform spectrum of the waveform.

In view of the frequency response, the sensitivity of the sensor mainly depends on the change in cavity length caused by the axial vibration, and the resonance frequency mainly depends on the length of $L_{2}$. By appropriately changing the length of $L_{2}$, the sensor can be designed to work at the required frequency. Therefore, we changed the length of $L_{2}$ to increase the working frequency of the sensor, and then tested whether the sensor worked normally from room temperature to 500 °C. At 500 °C, a 5 g acceleration was applied at a frequency of 200 Hz, and the vibration signal is shown in Figure 9.

**Figure 10:**
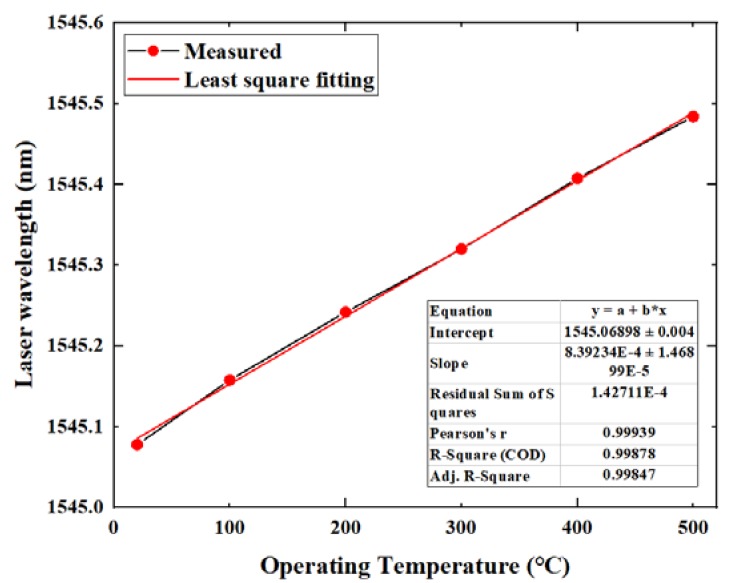
The wavelength of the phase offset point used in the test.

At different temperatures, temperature drift occurred in the sensor due to the influence of thermal expansion. Adjusting the wavelength of the tunable laser according to the temperature can eliminate the effect of this temperature drift. The wavelength of the phase offset point determined during the test is shown in Figure 10. We know that the temperature coefficient of the sensor was only 0.84 pm/°C. That is because the sensor has an all-silica structure, and the thermal expansion coefficient of the silica material is low. Therefore, the sensor has the advantage of temperature insensitivity.

## 4. Conclusions

We proposed a novel in-line fiber optic FP sensor for high-temperature vibration measurement. We constructed an FP cavity and a mass on single-mode fibers, and together they were inserted into a hollow silica glass tube to form a vibration sensor. The radial dimension of the sensor was less than 500 μm, where the FP cavity was constructed by fusing an HST at the end faces of two optical fibers, and the mass was constructed by fusing another type of HST on one of the optical fibers. When vibration occurs, the vibration of the mass causes changes of the FP cavity length, and the changes in cavity length can directly respond to changes in vibration. Adjusting the welding parameters can achieve stable welding without loss of light intensity. Adjusting the inside and outside diameter of the HST and the position of each welding point can stabilize the vibration measurement. We tested the sensor by using a vibration exciter and a light intensity demodulation system. In our test, the sensor had a resonance frequency of 165 Hz. The voltage sensitivity of the sensor system was about 11.57 mV/g and the nonlinearity was about 2.06%. The sensor has the advantages of high light intensity, small size, simple manufacturing process, and low cost. With its all-silica structure, the sensor has the prospect of measuring vibration in high-temperature environments. The sensor could work normally when the temperature was below 500 °C, and the drift of the phase offset point with temperature was 0.84 pm/°C.

## Figures and Tables

**Table 1:** The parameters of all hollow silica glass tubes (HSTs) and fibers used in the fabrication.

Name	Model Code	Inner Diameter (μm)	Outer Diameter (μm)
$\mathrm{Hollow}\;\mathrm{silica}\;\mathrm{glass}\;\mathrm{tubes}\;S_{1}$ $,\;S_{3}$ $,\;S_{4}$	YN135340, Yongnian Ruipu Chromatogram Equipment Co., Ltd. Yongnian, China	135	340
Hollow silica glass tube $S_{2}$	YN065125, Yongnian Ruipu Chromatogram Equipment Co., Ltd. Yongnian, China	65	125
Hollow silica glass tube $S_{5}$	YN350450, Yongnian Ruipu Chromatogram Equipment Co., Ltd. Yongnian, China	350	450

**Table 2:** The welding parameters for all welds.

Welding Mode	Power (W)	Time (ms)
Electrical discharge	4.3	200
Heating area A	10.2	800
Heating area B	12.8	800
